# Supplementary material for: Comparing alternatives to the fixed degree sequence model for extracting the backbone of bipartite projections
Source: Sci Rep. 2021 Dec 14;11:23929. doi: 10.1038/s41598-021-03238-3 (PMC8671427; doi:10.1038/s41598-021-03238-3)
Supplement: Supplementary file 1 — Supplementary Information. [file 41598_2021_3238_MOESM1_ESM.pdf]

Supplementary Information for:
"Comparing Models for Extracting the Backbone of Bipartite Projections"
Zachary P. Neal, Rachel Domagalski, & Bruce Sagan

S1 Probability Mass Functions of projection edge weights under ensemble backbone models

In the subsections below, we derive the probability mass functions of P^*_{ij} used by ensemble backbone models to evaluate the statistical significance of the weight of edge P_{ij} in a bipartite projection. We use the following notation:

- Let $\mathbf{B}$ be an $m \times n$ bipartite matrix, with a vector of row sums $R = (r_1, \ldots, r_m)$, a vector of column sums $C = (c_1, \ldots, c_n)$, and f cells containing a 1. So

$$f = \sum_{i=1}^{m} r_i = \sum_{j=1}^{n} c_j.$$

- Let $\mathcal{B}^{\mathsf{M}}$ be the ensemble of all $m \times n$ matrices $\mathbf{B}^* = (B^*_{ij})$ that obey the constraints of the respective model. In all models, the probability distribution on $\mathcal{B}^{\mathsf{M}}$ is uniform except in the stochastic case.
- Let P^*_{ij} be a random variable equal to $(\mathbf{B}^*\mathbf{B}^{*T})_{ij}$ for all $\mathbf{B}^* \in \mathcal{B}^{\mathsf{M}}$. Note that we have

$$P^*_{ij} = B^*_{i1}B^*_{j1} + B^*_{i2}B^*_{j2} + \cdots + B^*_{in}B^*_{jn}. \tag{1}$$

S1.1 Fixed Fill Model (FFM)

Let the *fixed fill model* constrain all $\mathbf{B}^* \in \mathcal{B}^{\mathsf{FFM}}$ to contain the same number of 1s (i.e. fill) as $\mathbf{B}$.

Theorem S1.1. *Under the fixed fill model, the distribution of P^*_{ij} for $i \neq j$ satisfies*

$$\Pr(P^*_{ij} = k) = \frac{\binom{n}{k} \sum_r 2^{n-k-r} \binom{n-k}{r} \binom{(m-2)n}{f-n-k+r}}{\binom{mn}{f}}. \tag{2}$$

Proof. For the denominator we need to compute the cardinality $\#\mathcal{B}^{\mathsf{FFM}}$. If $\mathbf{B}^* \in \mathcal{B}^{\mathsf{FFM}}$ then $\mathbf{B}^*$ has mn entries of which f must be chosen to be ones. So

$$\#\mathcal{B}^{\mathsf{FFM}} = \binom{mn}{f}.$$

For the numerator, suppose $P^*_{ij} = k$. We see from equation (1) that there are exactly k columns c where $B^*_{ic} = B^*_{jc} = 1$. There are $\binom{n}{k}$ ways to choose these columns. Now define the following parameters:

$$p = \text{number of columns } c \text{ where } B^*_{ic} = 1 \text{ and } B^*_{jc} = 0,$$
$$q = \text{number of columns } c \text{ where } B^*_{ic} = 0 \text{ and } B^*_{jc} = 1,$$
$$r = \text{number of columns } c \text{ where } B^*_{ic} = 0 \text{ and } B^*_{jc} = 0.$$

The number of ways to pick the columns counted by these parameters from the $n-k$ columns which do not contains ones in both rows is the trinomial coefficients $\binom{n-k}{p,q,r}$. Now we have used $2k+p+q$ ones in rows i and j. So there are $f-2k-p-q$ left to distribute to the remaining $m-2$ rows. And these rows have $(m-2)n$ entries. So the number of possibilities for these remaining ones is $\binom{(m-2)n}{f-2k-p-q}$. Thus the total number of choices from this and the previous paragraph is

$$\begin{aligned}\binom{n}{k}\sum_{p+q+r=n-k}\binom{n-k}{p,q,r}\binom{(m-2)n}{f-2k-p-q} &= \binom{n}{k}\sum_{p+q+r=n-k}\binom{n-k}{r}\binom{n-k-r}{p}\binom{(m-2)n}{f-n-k+r}\\ &= \binom{n}{k}\sum_{r}\binom{n-k}{r}\binom{(m-2)n}{f-n-k+r}\sum_{p}\binom{n-k-r}{p}\\ &= \binom{n}{k}\sum_{r}2^{n-k-r}\binom{n-k}{r}\binom{(m-2)n}{f-n-k+r}\end{aligned}$$

as desired. □

For even modestly large $\mathbf{B}$, computing equation (2) involves values larger than can be handled by some programs. In practice, we use logs to make these computations practical.

We now show that the sum in the numerator of this probability is related to the famous Jacobi orthogonal polynomials. This sum is a terminating hypergeometric series. Given a real number a and a nonnegative integer r the corresponding *Pochhammer symbol* or *rising factorial* is

$$(a)_r = a(a+1)(a+2)\cdots(a+r-1).$$

Note that if a is an integer with $-r < a \le 0$ then $(a)_r = 0$ because the product contains 0 as a factor. Given real numbers $a_1, a_2, \ldots, a_p$ and $b_1, b_2, \ldots, b_q$ as well as a variable z, the corresponding *hypergeometric series* is

$${}_pF_q\left[\begin{matrix} a_1 & a_2 & \ldots & a_p \\ b_1 & b_2 & \ldots & b_q\end{matrix}\,; z\right] = \sum_{r\ge 0}\frac{(a_1)_r(a_2)_r\cdots(a_p)_r}{(b_1)_r(b_2)_r\cdots(b_q)_r}\frac{z^r}{r!}.$$

Note that if any of the a_i are negative integers then, because of the remark above, this series will terminate and become a polynomial in z.

To convert a binomial coefficient into Pochhammer symbols, we write

$$\begin{aligned}\binom{n}{r} &= \frac{(n)(n-1)\cdots(n-r+1)}{r!}\\ &= \frac{(-1)^r(-n)(-n+1)\cdots(-n+r-1)}{(1)_r}\\ &= \frac{(-1)^r(-n)_r}{(1)_r}.\end{aligned}$$

The following identity will also be useful

$$\begin{aligned}(a)_{b+r} &= (a)(a+1)\cdots(a+b-1)\times(a+b)(a+b+1)\cdots(a+b+r-1)\\ &= (a)_b(a+b)_r.\end{aligned}$$

We now return to the sum in the numerator of equation (2). We will ignore the factor of 2^{n-k} since it is constant with respect to the sum and so can be pulled outside. For simplicity of calculation we will also use the substitutions

$$s = (m-2)n, \qquad t = f - n - k.$$

Thus we have

$$\begin{aligned}
\sum_r 2^{-r}\binom{n-k}{r}\binom{(m-2)n}{f-n-k+r} &= \sum_r \binom{n-k}{r}\binom{s}{t+r}(1/2)^r \\
&= \sum_r \frac{(-1)^r(k-n)_r}{(1)_r}\cdot\frac{(-1)^{t+r}(-s)_{t+r}}{(1)_{t+r}}(1/2)^r \\
&= (-1)^t \sum_r \frac{(k-n)_r(-s)_t(-s+t)_r}{(1)_t(t+1)_r}\frac{(1/2)^r}{(1)_r} \\
&= \frac{(-1)^t(-s)_t}{(1)_t}\sum_r \frac{(k-n)_r(-s+t)_r}{(t+1)_r}\frac{(1/2)^r}{r!} \\
&= \binom{s}{t}\ {}_2F_1\left[\begin{matrix} k-n & -s+t \\ & t+1 \end{matrix};\frac{1}{2}\right]
\end{aligned}$$

We are indebted to Marko Petkovšek [personal communication] for pointing out that this ${}_2F_1$ is, up to a factor, a specialization of a Jacobi polynomial. Given a nonnegative integer ℓ and real numbers α, β the associated *Jacobi polynomial* is

$$P_\ell^{(\alpha,\beta)}(z) = \binom{\alpha+\ell}{\ell}\ {}_2F_1\left[\begin{matrix} -\ell & \ell+\alpha+\beta+1 \\ & \alpha+1 \end{matrix};\frac{1-z}{2}\right]$$

To make these ${}_2F_1$ polynomials agree we can let $\ell = n-k$, $\alpha = t = f-n-k$,

$$\beta = -s + t - (\ell + \alpha + 1) = k - (m-1)n - 1$$

and $z = 0$. With these substitutions we get

$$\sum_r 2^{-r}\binom{n-k}{r}\binom{(m-2)n}{f-n-k+r} = \frac{\binom{(m-2)n}{f-n-k}}{\binom{f-2k}{n-k}}\ P_{n-k}^{(f-n-k,\ k-(m-1)n-1)}(0).$$

S1.2 Fixed Row Model (FRM)

Let the *fixed row model* constrain all $\mathbf{B}^* \in \mathcal{B}^{\mathsf{FRM}}$ to have the same row sums as $\mathbf{B}$.

Theorem S1.2. *Under the fixed row model, the distribution of P^*_{ij} for $i \neq j$ is hypergeometric and satisfies*

$$\Pr(P^*_{ij} = k) = \frac{\binom{r_j}{k}\binom{n-r_j}{r_i-k}}{\binom{n}{r_i}}.$$

Proof. The total number of ways to pick r_i of the n columns for ones in the ith row and r_j of the n columns for ones in the jth row is

$$\binom{n}{r_i}\binom{n}{r_j} = \binom{n}{r_i}\frac{n!}{r_j!(n-r_j)!}. \tag{3}$$

So that will go in the denominator of the desired probability.

For the numerator we follow the same line of reasoning as in the previous proof, where the parameters therein can be expressed as

$$\begin{aligned} p &= r_i - k, \\ q &= r_j - k, \\ r &= n - r_i - r_j + k. \end{aligned}$$

So we have a total of

$$\binom{n}{k}\binom{n-k}{p,q,r} = \frac{n!}{k!(r_i-k)!(r_j-k)!(n-r_i-r_j+k)!} \tag{4}$$

choices.

Dividing equation (4) by (3) and cancelling $n!$ gives

$$\Pr(P^*_{ij} = k) = \frac{\dfrac{r_j!}{k!(r_j-k)!}\cdot\dfrac{(n-r_j)!}{(r_i-k)!(n-r_i-r_j+k)!}}{\dbinom{n}{r_i}} = \frac{\dbinom{r_j}{k}\dbinom{n-r_j}{r_i-k}}{\dbinom{n}{r_i}}.$$

as desired. □

S1.3 Distribution of projection edge weights under the Fixed Column Model (FCM)

Let the *fixed column model* constrain all $\mathbf{B}^* \in \mathcal{B}^{\mathsf{FCM}}$ to have the same column sums as $\mathbf{B}$.

Let $X_1, \ldots, X_n$ be independent Bernoulli random variables. Let the probability of success for X_i be

$$\Pr(X_i = 1) = p_i.$$

The random variable

$$X = X_1 + \cdots + X_n \tag{5}$$

is said to have the *Poisson binomial distribution* with parameters $p_1, \ldots, p_n$.

Theorem S1.3. *Under the fixed column model, the distribution of P^*_{ij} for $i \neq j$ is Poisson binomial with parameters*

$$p_1 = \frac{c_1(c_1-1)}{m(m-1)},\ p_2 = \frac{c_2(c_2-1)}{m(m-1)},\ \ldots,\ p_n = \frac{c_n(c_n-1)}{m(m-1)}.$$

Proof. The B^*_{ik} are all either zero or one and are independent in different columns when only the column sums are fixed. So as k varies, the products $B^*_{ik}B^*_{jk}$ are independent Bernoulli random variables. Comparing equations (1) and (5), we see that the distribution of P^*_{ij} is Poisson binomial.

If column k has column sum $c = c_k$ then all zero-one vectors with sum c are equally likely for that column of $\mathbf{B}^*$. So there are $\binom{m}{c}$ possible kth columns. The number of ways to have a success is the number of possible columns which have ones in both positions i and j where $i \neq j$. So the number of choices is the number of ways to choose the remaining $c-2$ ones in that column from the other $m-2$ positions, that is, $\binom{m-2}{c-2}$. Thus

$$p_k = \Pr(B^*_{ik}B^*_{jk} = 1) = \frac{\binom{m-2}{c-2}}{\binom{m}{c}} = \frac{c(c-1)}{m(m-1)}$$

which finishes the demonstration. □

S1.4 Stochastic Degree Sequence Model (SDSM)

In the *stochastic degree sequence model*, $\mathcal{B}^{\text{SDSM}}$ consists of all binary $m \times n$ matrices. A method is then chosen to generate probabilities p^*_{ik}. Finally, matrices $\mathbf{B}^* \in \mathcal{B}^{\text{SDSM}}$ are generated using these probabilities for independent Bernoulli trials, where B^*_{ik} is filled with a one with probability p^*_{ik} and zero otherwise.

Theorem S1.4. *Under the stochastic degree sequence model, the distribution of P^*_{ij} for $i \neq j$ is Poisson binomial with parameters*

$$p_1 = p^*_{i1}p^*_{j1},\ \ldots,\ p_n = p^*_{in}p^*_{jn}.$$

Proof. The fact that the distribution is Poisson binomial follows immediately from the independence assumption on the $\Pr(B^*_{ik})$ and equation (1). Furthermore, the probability that the kth variable is one is

$$p_k = \Pr(B^*_{ik}B^*_{jk} = 1) = \Pr(B^*_{ik} = 1)\Pr(B^*_{jk} = 1) = p^*_{ik}p^*_{jk}.$$

So we are done. □

S2 Familywise error rates in backbone extraction

When testing the hypothesis that an observed statistic s is different from what would be expected at random (i.e. under a given null model), the researcher must specify a significance level α. The researcher then computes the probability p of observing a value greater than or equal to s under the null model. The null hypothesis is rejected and the alternative hypothesis is supported if $p < \alpha$. When only one hypothesis is being tested, this procedure ensures a Type-I error rate – a false positive, or the risk of rejecting the null hypothesis when it is true – of α.

In the context of backbone extraction, the 'statistic' s is the number of co-occurrences between two agent nodes or the edge weight in the bipartite projection, and the 'null model' is defined by the chosen bipartite ensemble backbone models. When deciding whether a given edge should be included in the backbone, the researcher is testing a single hypothesis where the null hypothesis is that the edge's

weight is no stronger than would be expected under the null model. If the null hypothesis is rejected, then the edge is included in the backbone. Committing a Type-I error in this context results in including the edge in the backbone when it should be excluded (i.e. a false positive).

When multiple independent hypotheses are tested simultaneously, the Type-I error rate is inflated. Specifically, the familywise error rate $\bar{\alpha}$ – the risk of making one or more Type-I errors – is $1-(1-\alpha)^t$, where t is the number of independent tests. For example, if the Type-I error rate for each hypothesis test is $\alpha = 0.05$, and $t = 100$ independent tests are performed, then $\bar{\alpha} = 1-(1-0.05)^{100} = 0.995$. That is, it is virtually guaranteed that at least one Type-I error will be committed in these 100 hypothesis tests. Because extracting a backbone requires the researcher to conduct a hypothesis test for every edge (with non-zero weight) in the network, backbone extraction nearly *always* involves testing multiple independent hypotheses.

Many different procedures exist for controlling $\bar{\alpha}$ when multiple independent hypothesis tests are conducted. All of these procedures involve using a corrected significance level α^* for each hypothesis test so that $\bar{\alpha}$ is maintained at the desired tolerance for Type-I error. The simplest but also most conservative approach is the Bonferroni correction, which defines $\alpha^* = \frac{\alpha}{t}$. Other less conservative and more powerful corrections include the Holm-Bonferroni correction [Holm, 1979] which has been used to extract the backbone of a political network [Aref and Neal, 2021], and the False Discovery Rate [FDR; Benjamini and Hochberg, 1995] which has been used to extract the backbones of movie rating and international trade networks [Saracco et al., 2017]. These correction procedures, as well as several others, are available in the `backbone` package we use to extract backbones in our studies [Domagalski et al., 2021].

Using one of these procedures to control $\bar{\alpha}$ is usually appropriate when extracting the backbone of a bipartite projection. Doing so is often straightforward because (1) many backbone models we consider (FRM, FCM, FFM, SDSM) yield exact p-values, and (2) the `backbone` package we use to extract backbones in our studies implements several different methods for correcting α^* and thus controlling $\bar{\alpha}$. However, for reasons we describe below, it is computationally infeasible to control $\bar{\alpha}$ when extracting backbones using FDSM. While this represents a significant limitation to using FDSM backbones in practice, and is a key reason we are seeking alternatives, this is not a problem for our studies. Within each of our studies, the rate of Type-I error inflation is identical for all backbones, which means that uncorrected FDSM backbones can be compared to uncorrected non-FDSM backbones.

S2.1 Controlling FWER in FDSM backbones

It is computationally infeasible to control $\bar{\alpha}$ when the backbone is extracted from a bipartite projection using FDSM. The challenge arises because each edge's p-value is estimated via a Monte Carlo procedure, and estimating these p-values with sufficient precision and confidence requires an impractically large number of Monte Carlo trials. In this section, we describe one way to estimate the required number of trials, and illustrate why controlling $\bar{\alpha}$ in FDSM backbones is impractical.

Because the associated probability mass function is unknown, when using FDSM to extract a backbone, the p-value of a given edge's weight (i.e. the probability that the same or larger edge weight would be observed under the null model) is estimated via Monte Carlo methods. Following N Monte Carlo trials in which a projection P^* is constructed from a random $\mathbf{B}^* \in \mathcal{B}^{\text{FDSM}}$, the p-value of the edge between i and j is

$$p_{ij} = \frac{\text{number of trials where } P^*_{ij} \geq P_{ij}}{N}.$$

Therefore, the estimation of p_{ij} is equivalent to estimating a proportion from a sample.

Determining the sample size required to estimate a proportion from a sample with a given error tolerance is a well-studied problem in statistical inference, under the heading of power analysis. Fleiss et al. [2013] show that the required minimum sample N to determine whether an estimated proportion P_1 differs from a hypothesized proportion P_0, with a Type-I error rate of ϵ_1 and Type-II error rate of ϵ_2, is

$$N \geq \left[\frac{z_{\epsilon_1}\sqrt{P_0\left(1-P_0\right)}+z_{\epsilon_2}\sqrt{P_1\left(1-P_1\right)}}{P_1-P_0}\right]^2,$$

where z represents the critical value corresponding to ϵ_1 or ϵ_2 in the standard normal distribution. Note that the Type-II error rate is the opposite of the Type-I error rate, the risk of failing to rejecting the null hypothesis when it is, in fact, false (i.e. a false negative). In the backbone context, committing a Type-II error results in excluding an edge from the backbone when it should be included. They further recommend performing a minor correction to arrive at a final estimate N'

$$N' \geq N + \frac{1}{|P_1-P_0|}.$$

With a small adaptation to their first expression, we can use these to estimate the required number of FDSM Monte Carlo trials. We wish to use it to determine the required minimum number of Monte Carlo samples N to determine whether an edge's estimated p-value p_{ij} differs from our corrected significance level α^*. Accordingly, we can re-write the expression given by Fleiss et al. [2013] as:

$$N \geq \left[\frac{z_{\epsilon_1}\sqrt{\alpha^*\left(1-\alpha^*\right)}+z_{\epsilon_2}\sqrt{p_{ij}\left(1-p_{ij}\right)}}{p_{ij}-\alpha^*}\right]^2.$$

Two examples serves to illustrate how this expression implies that an impractically large number of Monte Carlo trials will be required under even modest assumptions. Suppose we are using FDSM to extract the backbone from a projection of a 100 agent $\times$ 1000 artifact bipartite network, and we wish to maintain a familywise error rate of $\bar{\alpha} = 0.05$. If we assume that our bipartite projection will be dense (hence the need for extracting its backbone) and will not contain any zero-weight edges, then we must conduct $\frac{100(100-1)}{2} = 4950$ independent hypothesis tests. Using the Bonferroni correction for simplicity of illustration, this implies a corrected two-tailed significance level of $\alpha^* = \frac{0.05/4950}{2} \approx 0.000005$ for each test. Further, assume that we are willing to tolerate a 5% risk of incorrectly including an edge (i.e. Type-I error, $\epsilon_1 = 0.05$), and a 5% risk of incorrectly excluding an edge (i.e. Type-II error, $\epsilon_2 = 0.05$), because both types of errors are equally problematic for graphs.

Under these assumptions, we can consider two scenarios. First, we can determine how many (additional) trials are necessary to make a decision about an edge whose statistical significance appears unambiguous after some number of initial trials. When it appears that $p_{ij} = 0$, this represents a 'best case scenario' in which it should be relatively easy to reach a decision about whether the edge should be included in

the backbone. We can compute the required number of trials as:

$$N \geq \left[\frac{z_{.05}\sqrt{0.000005\,(1-0.000005)} + z_{.05}\sqrt{0\,(1-0)}}{0-0.000005}\right]^2$$

$$N \geq \left[\frac{1.64\sqrt{0.000005\,(1-0.000005)} + 1.64\sqrt{0\,(1-0)}}{0-0.000005}\right]^2$$

$$N \geq 535\ 695 \text{ (initial estimate)}$$

$$N' \geq 535\ 695 + \frac{1}{|0-0.000005|}$$

$$N' \geq 733\ 695 \text{ (adjusted estimate)}$$

Under this best case scenario, at least 733,695 Monte Carlo trials are required to reach a decision given our familywise error rate and tolerances for Type-I and Type-II errors. Recall that each Monte Carlo trial requires sampling one $\mathbf{B}^* \in \mathcal{B}^{\text{FDSM}}$ using the curveball algorithm, then multiplying $\mathbf{B}^*$ by its transpose. Although the running time of these two operations is relatively fast (approximately 0.07 seconds on the system we use to evaluate running times in Study 1), performing the required number of trials under this best case scenario would take around 14 hours.

Second, consider a more realistic scenario in which, after some initial number of trials, an edge's statistical significance is more ambiguous because p_{ij} is near α^*. For the sake of illustration, consider an edge whose p-value we initially estimate as $p_{ij} = 0.0000038$, which appears smaller than $\alpha^* = 0.000005$, but is close and therefore riskier. In this case,

$$N \geq 29\ 845\ 088 \text{ (initial estimate)}$$

$$N' \geq 30\ 637\ 088 \text{ (adjusted estimate)}$$

Under this more realistic scenario, where the edge's statistical significance is not unambiguous, over 30 million Monte Carlo trials are required to reach a decision given our error tolerance. This would require a running time of approximately 25 days.

References

Sture Holm. A simple sequentially rejective multiple test procedure. *Scandinavian journal of statistics*, pages 65–70, 1979.

Samin Aref and Zachary P. Neal. Identifying hidden coalitions in the us house of representatives by optimally partitioning signed networks based on generalized balance. *Scientific reports*, 2021.

Yoav Benjamini and Yosef Hochberg. Controlling the false discovery rate: a practical and powerful approach to multiple testing. *Journal of the Royal statistical society: series B (Methodological)*, 57 (1):289–300, 1995. 10.1111/j.2517-6161.1995.tb02031.x.

Fabio Saracco, Mika J Straka, Riccardo Di Clemente, Andrea Gabrielli, Guido Caldarelli, and Tiziano Squartini. Inferring monopartite projections of bipartite networks: An entropy-based approach. *New Journal of Physics*, 19(5):053022, 2017.

Rachel Domagalski, Zachary P Neal, and Bruce Sagan. backbone: An R package for extracting the backbone of bipartite projections. *PloS One*, 16(1):e0244363, 2021.

Joseph L Fleiss, Bruce Levin, and Myunghee Cho Paik. *Statistical methods for rates and proportions*. John Wiley & Sons, 2013.
